# Deep Convolutional Neural Networks-Based Automatic Breast Segmentation and Mass Detection in DCE-MRI

**DOI:** 10.1155/2020/2413706

**Published:** 2020-05-05

**Authors:** Han Jiao, Xinhua Jiang, Zhiyong Pang, Xiaofeng Lin, Yihua Huang, Li Li

**Affiliations:** ^1^School of Electronics and Information Technology, Sun Yat-sen University, Guangzhou 510006, China; ^2^Department of Medical Imaging, Sun Yat-sen University Cancer Center, State Key Laboratory of Oncology in South China, Collaborative Innovation Center for Cancer Medicine, Guangzhou 510060, China

## Abstract

Breast segmentation and mass detection in medical images are important for diagnosis and treatment follow-up. Automation of these challenging tasks can assist radiologists by reducing the high manual workload of breast cancer analysis. In this paper, deep convolutional neural networks (DCNN) were employed for breast segmentation and mass detection in dynamic contrast-enhanced magnetic resonance imaging (DCE-MRI). First, the region of the breasts was segmented from the remaining body parts by building a fully convolutional neural network based on U-Net++. Using the method of deep learning to extract the target area can help to reduce the interference external to the breast. Second, a faster region with convolutional neural network (Faster RCNN) was used for mass detection on segmented breast images. The dataset of DCE-MRI used in this study was obtained from 75 patients, and a 5-fold cross validation method was adopted. The statistical analysis of breast region segmentation was carried out by computing the Dice similarity coefficient (DSC), Jaccard coefficient, and segmentation sensitivity. For validation of breast mass detection, the sensitivity with the number of false positives per case was computed and analyzed. The Dice and Jaccard coefficients and the segmentation sensitivity value for breast region segmentation were 0.951, 0.908, and 0.948, respectively, which were better than those of the original U-Net algorithm, and the average sensitivity for mass detection achieved 0.874 with 3.4 false positives per case.

## 1. Introduction

Breast cancer is one of the most common cancers amongst women worldwide [1]. Early diagnosis and treatment are proven to reduce the mortality rate [2]. Dynamic contrast-enhanced magnetic resonance imaging (DCE-MRI) is a more reliable tool for early detection of breast cancer than mammography and ultrasound [3]. The correct resolution of DCE-MRI image of the breast depends largely on the quality of visualization, operation experience, and the time needed for data analysis. Because manual analysis of MR series is time-consuming and error-prone, several specific systems have been developed to help radiologists detect and diagnose breast lesions, which greatly improves clinicians' work efficiency [4]. Although some computer-aided diagnosis (CAD) systems are currently used in the clinic, fully automatic detection of breast lesions is still an ongoing problem [5].

Generally, a DCE-MRI image of the breast also includes other organs such as the lung, heart, liver, and pectoral muscles. Separation of the breast region from other organs is necessary for further analysis. Manual segmentation of the breast region is tedious; therefore it is impractical to segment the entire breast region from a large number of diverse datasets. Automatic segmentation of the breast region reduces result bias and can accelerate data processing. Conventional breast region segmentation methods proposed in the literature are based on threshold, morphology, and fuzziness with specific connections between pixels. Thakran et al. segmented the outer and inner breast tissue by thresholding, morphological operation, and B-spline curve fitting [6]. Wu et al. [7] combined edge enhancement and edge linking with candidate evaluation to detect the chest-wall line in the sagittal plane. Jiang et al. [8] used dynamic programing combined with preprocessing to segment the breast region in fat-suppressed transverse DCE-MRI. While these traditional methods have shown good performance, the robustness of these algorithms is insufficient, in that they tend to fail in some specific data because they can only process the underlying information. In recent years, convolutional neural networks (CNNs) have been widely used in biomedical semantic segmentation such as FCN [9], SegNet [10], and U-Net [11]. These deep networks can automatically extract high-level features and complete pixel-wise segmentation. Xu et al. used a 2D U-Net for automatic breast region segmentation in DCE-MRI [12]. Adoui et al. built two fully CNNs based on SegNet and U-Net for breast tumor segmentation [13]. Building on the success of U-Net in medical image segmentation, this study additionally used U-Net++ [14] for comparison of DCE-MRI images for breast region segmentation. Our results indicated the effectiveness and accuracy of this method in biomedical segmentation tasks.

Because of the varying sizes, shapes, appearances, and densities of masses, CAD for breast mass detection is a challenging task [15, 16]. Conventional methods for breast mass detection mainly rely on threshold values [17] or mass templates [18] based on various kinds of filter operators. Huang et al. used multiscale Hessian-based analysis for breast mass detection [19], and Wang et al. detected breast masses based on Gestalt psychology [20]. These traditional detection algorithms are sensitive to image noise, and the hand-designed features are not adequately robust to blurred contrast and bias-field. Deep convolutional neural networks (DCNNs) have significantly outperformed traditional methods in recent years, owing to the strong feature expression ability that can further improve the detection accuracy. With the development of deep learning, DCNN has been widely used in medical image detection [21–23]. These CAD systems can be used to migrate between breast cancer and lung cancer. The application of a CAD system helps the radiologist as a second reviewer to evaluate screening medical images. A CAD system based on one of the most successful object detection frameworks—Faster RCNN [24]—could achieve high sensitivity with few false positive results on the datasets of mammograms and tomosynthesis [25, 26]. In the field of mammary MRI, the development of deep learning frameworks is limited, because only a small number of datasets are available. With this background, we incorporated Faster RCNN into our DCE-MRI datasets for detection of breast mass.

In this paper, DCNN-based frameworks were present for computer-aided segmentation of breast region and detection of breast masses. Breast region segmentation was first performed by U-Net++ to remove other organs except the breast region to eliminate interference. U-Net was also implemented as a comparison of segmentation framework. Given the limited amount of our datasets, data augmentation was used during training to reduce overfitting. Breast mass detection was implemented by training Faster RCNN with images preprocessed by segmentation network and labels annotated by radiologists. The proposed methods were validated by a 5-fold cross validation technique to obtain more reliable results than independent tests. Performance of the automatic segmentation was evaluated using similarity coefficients. The breast mass detection model was evaluated using sensitivity at the number of false positives per case.

## 2. Materials and Methods

### 2.1. Data

The dataset consisted of 75 women (mean age, 47 years; age range, 18–68 years) with histopathologically confirmed breast masses at the Sun Yat-sen University Cancer Center (Guangzhou, China). Patients with suspicious breast masses were recruited after they provided written informed consent. The Ethics Committee of Sun Yat-sen University Cancer Center approved the study. Patients were scanned in the prone position with the bilateral breast naturally hanging into the two holes of the coil. A 3.0 T superconductive magnetic system (Discovery 750, GE Healthcare) with a bilateral 8-channel phased-array breast-specific surface coil was used for the imaging. Standard imaging was performed, including axial fast spin echo (FSE) T1WI, and axial and sagittal FSE T2WI. Subsequently, diffusion-weighted images (DWI) were acquired in the axial planes. The DCE-MRI data were acquired using the VIBRANT-FLEX technique in the axial orientation, after one set of unenhanced baseline images were obtained using an MRI-specific automatic power injector (Medrad Inc., Pittsburgh, PA, USA) to inject 0.1 mmol/kg body weight contrast medium (gadopentetate dimeglumine; Magnevist, Bayer Schering Pharma, Berlin, Germany), with a hand venipuncture technique at a rate of 3 mL/s. Saline (10 mL at 3 mL/s) was then injected to wash the tube. Dynamic scanning was initiated by simultaneously pushing the high-pressure syringe button and the dynamic scan button. Eight postcontrast sets were acquired under the following scanning conditions: field of view, 32 cm; matrix, 320 × 320; section thickness without a gap, 1.4 mm; repetition time, 3.9 ms; echo time, 1.7 ms; and flip angle, 5°. The patients had not received any treatment before undergoing MRI. In this study, only mass-like masses showed strong contrast.

Diagnosis was confirmed following pathological analysis subsequent to core-needle biopsy or surgical excision, or lesion not changed at a minimum follow-up of 2 years defined as benign lesion.

All images were analyzed independently by two radiologists with ten years of experience in breast MRI. The images were assessed independently and any disagreements were resolved by achieving consensus. All lesions were assessed using the Breast Imaging Reporting and Data System (BI-RADS). BI-RADS category 1 (negative) and category 2 (benign) denote an essentially 0% likelihood of cancer. BI-RADS category 3 (probably benign) assessment is more intuitive and can be recommended in the case of a unique focal finding for which the likelihood of malignancy is ≥0% but ≤2%. BI-RADS category 4 (suspicious) and category 5 (highly suggestive of malignancy) describe MRI findings that are suspicious enough to warrant tissue diagnosis. BI-RADS category 6 (known biopsy-proven malignancy) describes MRI findings of biopsy-proven breast cancer for which surgical excision is recommended when clinically appropriate.

The objective of this study is to detect the mass. All lesions with a BI-RADS score greater than 1 were used in the dataset. According to statistics, the diameter of the mass ranges from 1.07 to 6.69 cm.

## 3. Methods

**Figure 1:**
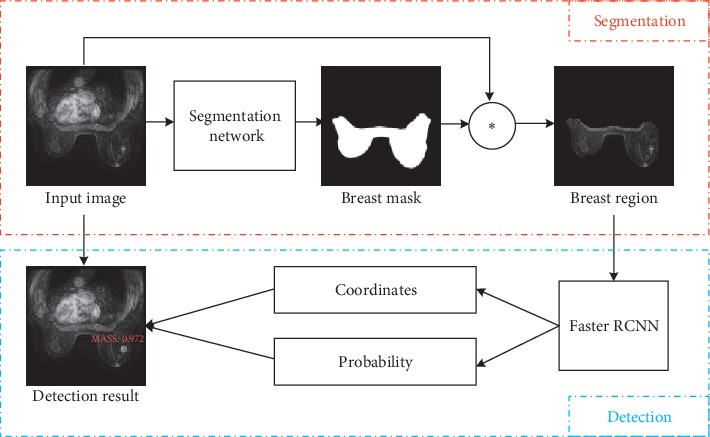
The framework of breast region segmentation and breast mass detection. The entire framework is divided into two parts. The upper part is image segmentation. Breast region mask is obtained by importing the image into segmentation network such as U-Net++ and U-Net. The breast region can be segmented by masking the input image. The following part is the target detection. The breast region image was input to Faster RCNN to obtain the location coordinates and probability of the mass.

Our fully automatic method consists of two parts: breast region segmentation and breast mass detection. Automatic segmentation of breast region is a challenging task because of large variations in breast shapes, sizes, image artifacts, and other noise-induced errors. Inspired by the success of DCNN models on object segmentation tasks, we implemented a U-Net++ framework for breast region segmentation, along with a U-Net framework for comparison. Next, we obtained images of the breast region without interference from other organs/body parts as the input for Faster RCNN to detect breast masses. Finally, the positions and sizes of the breast masses were identified. The whole framework of this proposed method is shown in Figure 1.

**Figure 2:**
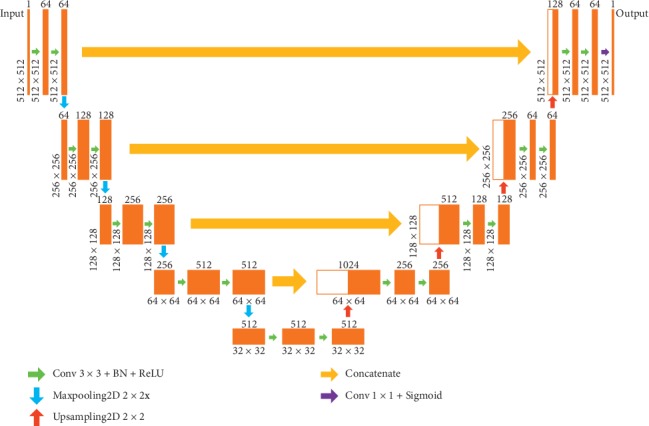
The architecture of U-Net. The network consists of two parts. The left half is feature extraction. After each pool layer, the scale of feature changes. There are five scales in total. The right half is upsampling. Every time the feature is upsampled, it will be fused with the same scale corresponding to the feature extraction part.

U-Net [11] was used in breast region segmentation for comparison, and its architecture is shown in Figure 2. One advantage of this method is its robustness even with small training data. The architecture consists of downsampling (left side) and upsampling (right side). The left side acts as an encoder and extracts features through the network. It contains a typical convolutional structure: two 3 × 3 convolution operations and one 2 × 2 max-pooling operation. Each convolution operation is followed by batch normalization (BN) and a rectified linear unit (ReLU). The right side acts as a decoder and also contains a typical architecture: a 2 × 2 upsampling operation and two 3 × 3 convolution operations, each followed by BN and a ReLU. Contracting paths are used to combine the high resolution feature maps with the upsampling outputs to accurately classify and locate each pixel. Every pixel of input image is classified as breast region or background. To evaluate the segmentation loss, we used the binary cross entropy (BCE) loss function.

(1) $$\begin{array}{c} \text{BCE Loss}=\;-\frac{1}{M}\sum_{i=1}^{M}\left(Y_{i}\cdot \;\log\left({\hat{Y}}_{i}\right)+{\left(1-Y\right)}_{i}\cdot \;\log\left(1-{\hat{Y}}_{i}\right)\right), \end{array}$$

where *Y* is the ground truth and $\hat{Y}$ is the predicted probability for all the *M* pixels.

**Figure 3:**
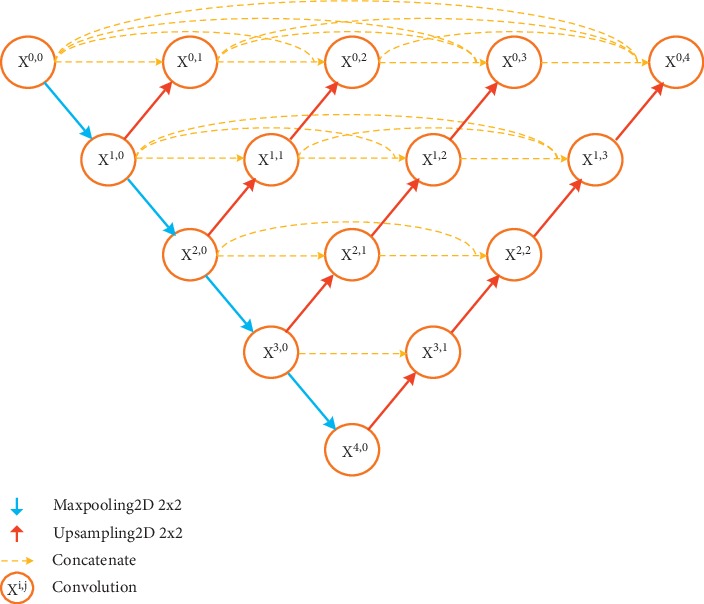
The architecture of U-Net++. Every *X*^*i*,*j*^ includes two 3 × 3 convolution operations, each followed by BN and a ReLU. Channels of the same scale are connected in a dense manner for gradient propagation.

U-Net++ [14] is an improved version of U-Net for biomedical image segmentation. For the skip connection of U-Net, U-Net++ adds modules to integrate the features of different levels through superposition. Further, to ensure gradient propagation, U-Net++ uses a deep supervision scheme that connects the middle module to the final output, finally forming a dense block structure as shown in Figure 3. The loss function is a combination of binary cross entropy and dice coefficient on each of the above four semantic levels, which is described as

(2) $$\begin{array}{c} \text{BCE\&Dice Loss}=\;-\frac{1}{N}\sum_{b=1}^{N}\left(\frac{1}{2}\cdot Y_{b}\cdot \;\log\left({\hat{Y}}_{b}\right)+\frac{2\cdot Y_{b}\cdot {\hat{Y}}_{b}}{Y_{b}+{\hat{Y}}_{b}}\right)\;, \end{array}$$

where *Y*_*b*_  and ${\hat{Y}}_{b}\;$ denote the flattened ground truths and the flattened predicted probabilities of the *b*^*th*^ image, respectively, and *N* indicates the batch size.

**Figure 4:**
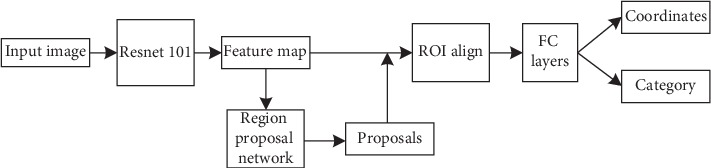
The architecture of Faster RCNN. Using ResNet-101 as the feature extraction network, the target location and probability can be obtained by processing the shared features.

The object detection network named Faster RCNN [24] is composed of two modules. The first module is a deep fully CNN that proposes regions and the second module is the detector that uses the proposed regions. Feature map is extracted from input image by using ResNet-101 [27]. ResNet-101 has been proven to perform well on classification tasks, which shows that it has good feature extraction ability. A region proposal network (RPN) considers the feature map as input and provides a set of rectangular object proposals as the output, each with an objectness score. The RPN structure in [24] is used in this paper without modification. The function of ROI Align is to map region of interest (ROI) areas of different sizes to feature maps of fixed sizes. ROI Align [28] can effectively solve the problem of misalignment caused by twice quantization in ROI Pooling. For the detection of large objects, the difference between the two schemes is rare. If there are more small objects in the picture to be detected, ROI Align is preferred, which is more accurate. In this paper, the masses of breast MRI image are taken as the research target, and the diameter of them is less than 48 pixels, which belongs to small targets. Therefore, ROI Align is selected for detection network. The feature is shared by RPN and fully connected (FC) layers. The structure is shown in Figure 4.

We computed four regression losses. *L*_reg_ was associated with predicted mass coordinates, widths, and height, and *L*_cls_ was the classification loss for the predicted mass probabilities. The ground truth labels are determined for each anchor as follows: If an anchor *i* overlaps with a mass with an intersection over union (IOU) greater than 0.7, then it is regarded as positive (*p*_*i*_^*∗*^=1). On the contrary, if an anchor *i* overlaps with a mass with an IOU less than 0.3, it is regarded as negative (*p*_*i*_^*∗*^=0). All other anchors do not contribute to the loss, and only positive anchors contribute to the regression loss. The final loss function for anchor *i* is defined as follows:

(3) $$\begin{array}{c} L\left(p_{i},\;t_{i}\right)=\frac{1}{N_{\text{cls}}}\sum_{i}L_{\text{cls}}\left(p_{i},\;p_{i}^{*}\right)+\;\frac{1}{N_{\text{reg}}}\sum_{i}L_{reg}\left(t_{i},\;t_{i}^{*}\right). \end{array}$$

Here, *t*_*i*_ is a vector representing the four parameterized coordinates of the predicted bounding box and *t*_*i*_^*∗*^ is the ground truth associated with a positive anchor. We used binary cross entropy loss for *L*_cls_ and smooth L1 loss for *L*_reg_.

### 3.1. Performance Evaluation

The performance of the proposed method for breast region segmentation was tested using the Dice similarity coefficient (DSC) [29], Jaccard coefficient [30], and segmentation sensitivity described by Udupa et al. [31], which are given by the following equations:

(4) $$\begin{array}{c} \text{Dice Coefficient DSC}=\;2\cdot \frac{\left|A\cap B\right|}{\left|A\right|+\left|B\right|},\\ \text{Jaccard Coefficient Jaccard}=\;\frac{\left|A\cap B\right|}{\left|A\right|+\left|B\right|-\left|A\cap B\right|}\;,\\ \text{Segmentation Sensitivity SS}=\frac{\left|A\cap B\right|}{\left|B\right|}\;, \end{array}$$

where *A* is the automatic segmentation result and *B* is the ground truth.

For target detection tasks, performance is measured by sensitivity with the average number of false positives per sample. In the task of breast mass detection, the sensitivity, also known as the true positive rate (TPR), represents the proportion of the number of detected masses to the number of all masses in the dataset. This is calculated using the true positive (TP), false negative (FN), and false positive (FP). It should be noted that true negative (TN) in target detection tasks is meaningless.

(5) $$\begin{array}{c} \text{True Positive Rate TPR}=\;\frac{\text{TP}}{\text{TP}+\text{FN}}. \end{array}$$

The receiver operating characteristic (ROC) curve was first invented by electronics and radar engineers during World War II to detect enemy vehicles on the battlefield, i.e., signal detection theory. Soon afterwards, it was introduced into psychology to detect signal perception. Since then, it has been introduced into the field of machine learning to evaluate classification and test results and is a very important and common statistical analysis method. However, the classical ROC method cannot solve the practical problem of evaluating target detection task on an image. In the 1970s, the concept of FROC (free-response ROC) was proposed, which allows the evaluation of arbitrary anomalies on each image. The FROC curve considers the number of false positives in each sample as the *x*-axis and the recall as the *y*-axis. The closer the curve to the upper left corner, the better the performance of the model.

## 4. Results

### 4.1. Breast Segmentation

Owing to the uncertainty of breast MRI, traditional methods cannot at times segment breast region very well. Therefore, in this study, we adopted the deep learning method for breast region segmentation.

**Figure 5:**
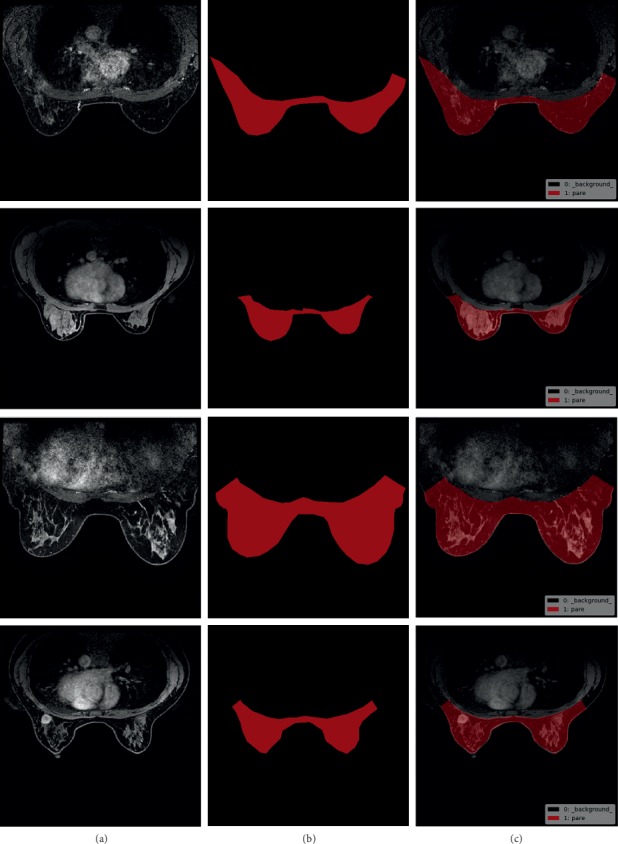
Samples of breast region segmentation dataset: (a) the original image, (b) the segmentation ground truth, and (c) (b) superimposed on (a). The pixel value of the breast region is set to one and the pixel value of the background area is set to zero during training of segmentation models.

**Figure 6:**
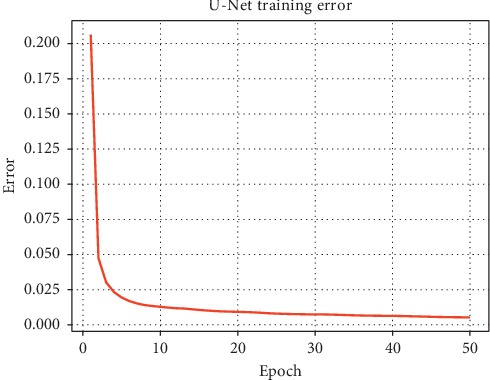
U-Net training error.

**Figure 7:**
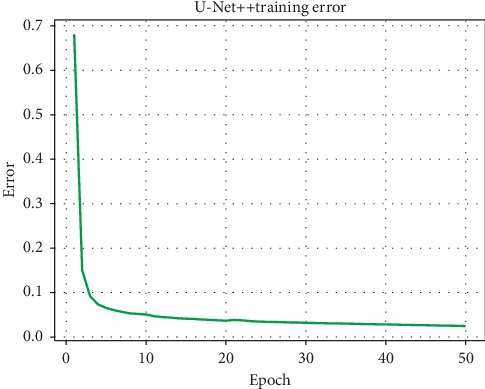
U-Net++ training error.

We proposed breast region segmentation by training a U-Net++ model, which removes interference from different DCE-MRI series external to the breast region. We used the dataset of DCE-MRIs from 75 patients. For the segmentation task, we created breast region labels for each patient (Figure 5). Because of the similarity in consecutive images in the DCE-MRI series, we allocated one breast region label for every 10 consecutive images. We employed patient-level, 5-fold cross validation. Each subset contains 15 cases of DCE-MRI. Moreover to illustrate the robustness of our algorithm, we trained our model on four subsets and validated it on the other subset. For data augmentation, the training sets were flipped horizontally. We trained the U-Net++ model and the U-Net model as comparison for 50 epochs using stochastic gradient descent (SGD) as the optimizer and used the last epoch to predict validation datasets. The batch size was set to five given the limitation of GPU memory. The training processes of U-Net and U-Net++ are shown in Figures 6 and 7, respectively, which show the final convergence of the networks.

**Figure 8:**
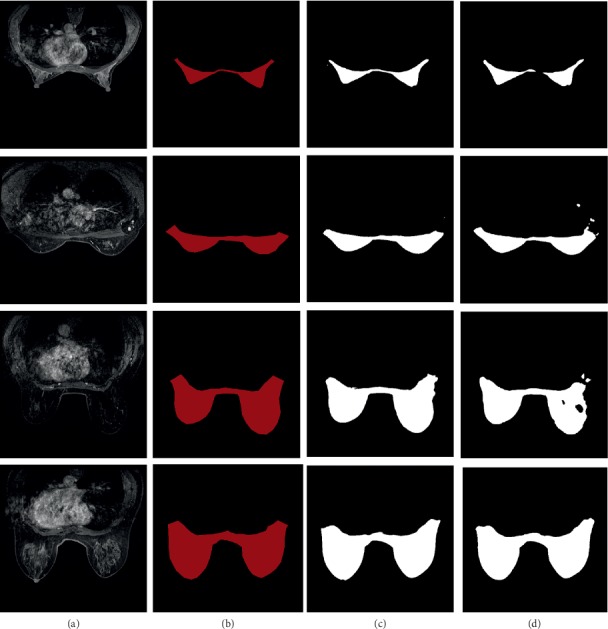
Samples of breast region segmentation result: (a), (b) source images and ground truths, respectively, (c) the breast region segmentation result of U-Net++, and (d) segmentation from U-Net.

The segmentation performance was validated by DSC, Jaccard, and segmentation sensitivity on the validation dataset. We computed performance values of U-Net++ for each validation image, and the average of them was 0.951, 0.908, and 0.948, respectively, which were better than those of U-Net. Evaluation results are provided in Tables 1 and 2. Exp 1 to 5 are experiments from 5-fold cross validation. The performance of each experiment result was evaluated, and mean values of the Dice coefficient (DSC), Jaccard coefficient, and segmentation sensitivity between segmentation results and ground truths from 15 different cases are calculated. The average performance was assessed. The breast region segmentation results on the validation dataset are shown in Figure 8. From the overall segmentation effect, the integrity and robustness of U-Net++ segmentation were better than those of U-Net.

### 4.2. Mass Detection

**Figure 9:**
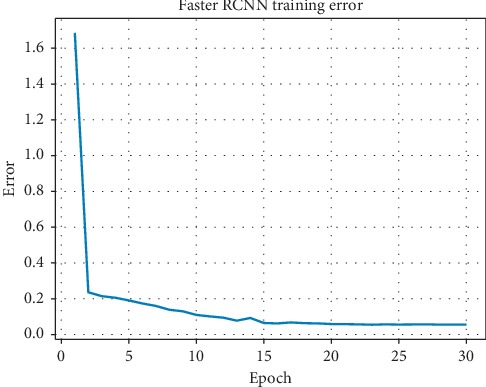
Faster RCNN training error.

Encouraged by the overall success of Faster RCNN and deep residual networks in natural images, we used them in our study of breast mass detection. To eliminate the impact of redundant information and external noise, we first used the well-trained U-Net++ model to preprocess images. After that, the training and validation dataset only contained information pertaining to the breast region. All the slices containing the mass were extracted, and the central coordinates and the width and height of the mass on the image were marked for training. In validation, the whole case was segmented and processed. The detection results obtained by the network were mapped to three-dimensional space, and the bounding boxes close to each other in the space would be merged into one candidate. Candidates would be evaluated as TP if the IOUs with ground truth were greater than 0.5 while FP if less than 0.5. We employed patient-level 5-fold cross validation. Each subset contains 15 cases of DCE-MRI. Further, to illustrate the robustness of our algorithm, we trained our model on 4 subsets and validated it based on the other remaining subset. In the training subset, we used 30 epochs in total with SGD optimization and a momentum of 0.9. Because of the limitation of GPU memory, the batch size parameter was set to 8. We used a weight decay of 5 × 10^−4^. The initial learning rate was 0.001 and multiplied by 0.1 every 10 epochs. Before training the network, the weights of pretrained ResNet-101 were loaded for transfer learning, which effectively accelerated convergence. The training process of the network is shown in Figure 9, which shows the final convergence of the network.

**Figure 10:**
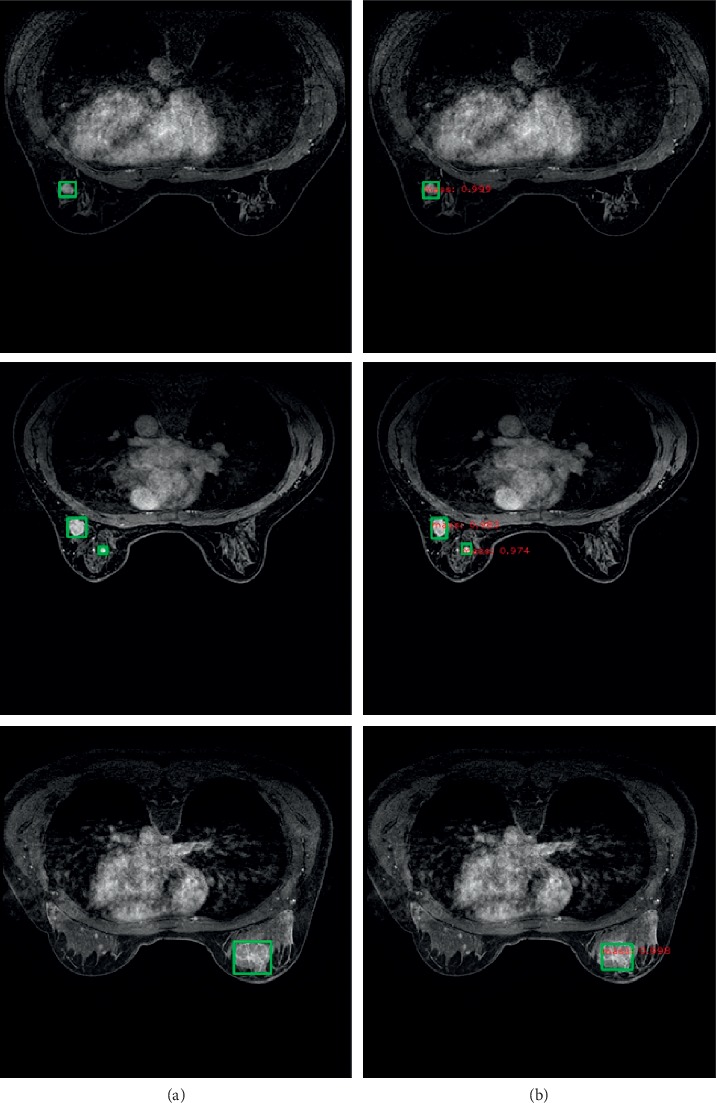
Ground truth and detection result: (a) the ground truth of breast mass detection and (b) the detection results of Faster RCNN, including target position, size, and probability.

**Figure 11:**
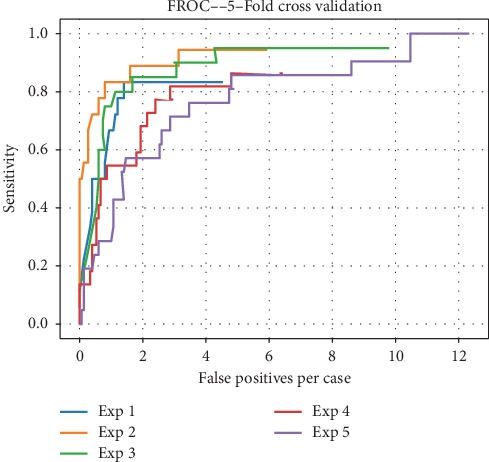
FROC of each experiment. The FROC curves of Exp 1 to Exp 5 are listed. Horizontal coordinates represent the average number of false positives per case. The vertical coordinates represent the sensitivity corresponding to the average number of false positives per case.

**Figure 12:**
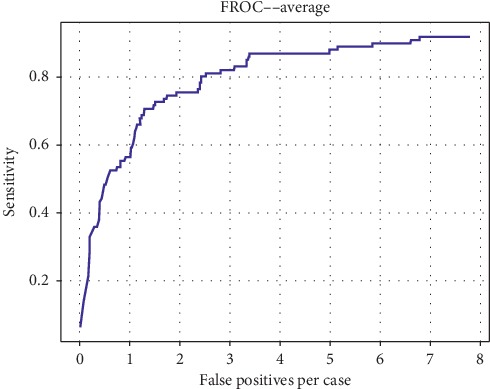
Average FROC for 5-fold cross validation.

We validated the Faster RCNN model based on the validation dataset and the average mass-level sensitivity achieved 0.874 at 3.4 false positives per case. The comparison of several detection results is shown in Figure 10. The network outputs the specific location and size information of different kinds of mass, as well as the probability. Regarding the 5-fold cross validation, Figure 11 shows the FROC curve of each experiment and Figure 12 shows the average FROC curve for 5-fold cross validation. The total size of the dataset is small so that it is probable that a few samples are obviously different from other cases, which affects the accuracy of the experimental results. Therefore, the cross validation method is used to make the experimental results more convincing in small datasets. The performance of the curves supports the proposed breast mass detection method as a useful tool to assist doctors with the diagnosis.

## 5. Discussion

We proposed a method based on DCNN to effectively segment breast region and detect breast masses on DCE-MRI images. The success of the proposed method was mainly dependent on two aspects. First, breast region segmentation is necessary because other organs outside the breast occupy most of the image area, which greatly interferes with detection of masses. U-Net++ is a fully convolutional network applicable to various biomedical segmentation problems. The network contains convolutional layers and max-pooling layers and does not have any fully connected layers. Specifically, there are dense blocks between skip connections, which can make full use of extracted features. Owing to the lack of datasets, 5-fold cross validation was adopted. We used our own data to train U-Net++ for 50 epochs, and the DSC, Jaccard, and segmentation sensitivity values obtained were 0.951, 0.908, and 0.948, respectively, which were better than those obtained with U-Net. The experimental results showed that U-Net++ segmentation of the breast region was more precise and complete than the U-Net segmentation. As seen in Figure 8, the segmentation of the end of the breast region was not very accurate, but the key areas were well preserved, which was helpful in mass detection. Second, Faster RCNN is a flexible generic object detection framework that is easily extended to biomedical detection tasks. This study used ResNet-101 as the backbone of Faster RCNN to extract feature map from the input image. Then, there were two parallel branches to share the feature map, bounding box regression, and classification. We used the well-trained U-Net++ model to preprocess the training dataset to eliminate interference. Before training the network, the weights of pretrained ResNet-101 were loaded for transfer learning, which effectively accelerated convergence. For the performance evaluation, Faster RCNN accurately found the position of the breast mass and provided corresponding confidence information. We used 5-fold cross validation, and the average sensitivity performance achieved 0.874 with 3.4 false positives per case. This meant that our results may have a potential value for early diagnosis and treatment of breast cancer, but more clinical cases are needed to carry out research for verification.

Our study has some limitations, but it can verify the feasibility of this method in our dataset. Future research includes detection from multiple directions as well as using 3D CNN for extracting 3D feature map. The dataset used in this paper is relatively small. We aim to establish a large-scale open dataset for convenient performance comparison of papers with different algorithms in future.

## 6. Conclusion

Herein, we presented an automatic method for breast region segmentation and mass detection based on DCNN in DCE-MRIs. Our method consists of the breast region segmentation by U-Net++ and the breast mass detection by Faster RCNN. The DCNNs were trained and validated in DCE-MRIs from Sun Yat-sen University Cancer Center and showed good performance. We believe that this method provides a powerful clinical tool to help doctors accurately and quickly diagnose breast masses. Future research will include the establishment of larger open data and the opinions of radiologists and surgeons in clinical practice.

## Figures and Tables

**Table 1:** Performance of breast region segmentation by U-Net on 5-fold cross validation.

Performance	Exp 1	Exp 2	Exp 3	Exp 4	Exp 5	Average
DSC	0.949	0.952	0.926	0.938	0.938	0.941
Jaccard	0.905	0.908	0.869	0.886	0.885	0.891
Segmentation sensitivity	0.947	0.960	0.933	0.946	0.921	0.941

**Table 2:** Performance of breast region segmentation by U-Net++ on 5-fold cross validation.

Performance	Exp 1	Exp 2	Exp 3	Exp 4	Exp 5	Average
DSC	0.959	0.960	0.945	0.948	0.942	0.951
Jaccard	0.921	0.924	0.897	0.904	0.894	0.908
Segmentation sensitivity	0.957	0.964	0.940	0.953	0.927	0.948

## Data Availability

The DCE-MRI data used to support the findings of this study were supplied by the Sun Yat-sen University Cancer Center (Guangzhou, China) under license and have not been made freely available because of patient privacy. If our dataset is useful to you, please contact the corresponding authors by e-mail.
